# Citrus Flavonoids-Coated Biogenic Nanoparticles for Controlling *Xanthomonas axonopodis* pv. *citri*: Antimicrobial Efficacy and NMR-Based Investigation of Bacterial Metabolic Reprogramming

**DOI:** 10.1021/acsabm.5c01809

**Published:** 2025-12-15

**Authors:** Gonzalo Garcia Delgado, Lyvia Malvestiti Cardoso Da Cunha, Thyerre Santana da Costa, Ljubica Tasic

**Affiliations:** Institute of Chemistry, Biological Chemistry Laboratory, Universidade Estadual de Campinas, UNICAMP, Campinas, SP 13083-970, Brazil

**Keywords:** flavonoid-functionalized biogenic nanoparticles, metabolic reprogramming in phytopathogens, sustainable nanomaterials for agriculture, NMR-based metabolomics, Citrus canker control alternatives, biogenic silver nanoparticles antimicrobial mechanism

## Abstract

Citrus canker, caused by the Gram-negative bacterium *Xanthomonas axonopodis* pv. *citri* poses a major threat to global citrus production, with increasing resistance to copper-based pesticides and antibiotics. This study presents a sustainable approach for bacterial control using biogenic silver nanoparticles (AgNPs) synthesized via green methods and functionalized with citrus peel-derived flavonoids, hesperidin and hesperetin. These flavonoid-coated nanoparticles—AgNP@HSD and AgNP@HST, respectively—were structurally characterized, confirming successful biofunctionalization and providing insight into flavonoid–nanoparticle interactions. Antimicrobial assays demonstrated potent inhibitory effects against *X. axonopodis* pv. *citri*, with both formulations outperforming streptomycin in terms of minimum inhibitory concentrations. Mechanistic investigations revealed distinct antimicrobial pathways: AgNP@HSD induced a broad stress response marked by elevated amino acid levels and extensive membrane damage, while maintaining intracellular ATP and NAD­(P)H levels, suggesting a metabolic adaptation strategy. In contrast, AgNP@HST triggered targeted oxidative stress, leading to significant depletion of energy and redox metabolites, indicating collapse of cellular homeostasis. This differential behavior is attributed to the higher intrinsic bioactivity of hesperetin, the aglycone form of hesperidin. Our findings underscore the potential of citrus flavonoid-coated biogenic nanoparticles as eco-friendly biomaterials for plant disease management. The integration of green nanotechnology with plant-derived metabolites offers a promising alternative to conventional agrochemicals for the control of citrus canker and other phytopathogenic threats.

## Graphical abstract

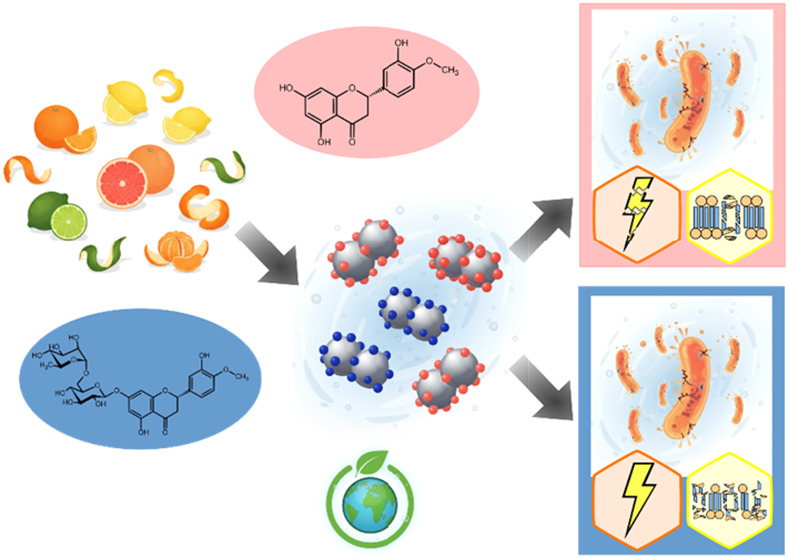

## Introduction

*Xanthomonas axonopodis* pv. *citri* is a phytopathogenic bacterium responsible for the citrus canker disease, an economically significant disease in citrus trees.^1–3^ Its ability to form biofilms, persist in the environment, and develop resistance to conventional treatments poses a major challenge to sustainable agricultural management.^4,5^ The use of windbreaks, copper sprays, and leafminer control for managing citrus canker,^6^ and efforts are focused on new alternatives to actual antibiotics.^7–12^

Nanotechnology has emerged as a powerful tool in the development of new antimicrobial agents.^13–17^ In particular, silver nanoparticles (AgNPs) are well-known for their broad-spectrum antibacterial activity, low tendency to induce resistance, and versatility in synthesis and surface modification.^13,18,19^ Recent advances have focused on the functionalized AgNPs using plant-derived compounds, which not only improve biocompatibility, but also allow for surface functionalization with bioactive molecules.^20–22^

Flavonoids, such as hesperidin and hesperetin, are natural polyphenolic compounds with recognized antioxidant and antimicrobial properties.^23–30^ When used to functionalize silver nanoparticles, these molecules can enhance the particles’ stability and bioactivity, potentially improving their effectiveness against phytopathogens. While demonstrating antibacterial efficacy is a critical first step, a deeper understanding of the underlying mechanism of action is essential for optimizing these nanoagents. Metabolomics, which provides a functional snapshot of cellular biochemistry, is a powerful systems-biology approach to elucidate these mechanisms by capturing the comprehensive metabolic response of an organism to a specific stressor.^31,32^ Two major platforms dominate the field of metabolomics. On the one hand, mass spectrometry (MS), often coupled with liquid (LC-MS) or gas chromatography (GC-MS), is the most popular choice.^33,34^ MS offers exceptional sensitivity, high specificity and resolution, and great versatility, making it ideal for discovering low-abundance biomarkers.^34,35^ On the other hand, Nuclear Magnetic Resonance (NMR) spectroscopy offers a different set of significant advantages. It is highly reproducible, requires minimal sample preparation, and can simultaneously detect and quantify a wide range of structurally diverse metabolites in a single, nondestructive experiment.^34,36,37^ Unlike MS, which excels at sensitive detection of specific molecules, NMR provides a more quantitative and unbiased view of the most abundant metabolites and can also yield insights into the structure of larger molecules like lipoproteins.^34^ By monitoring shifts in crucial metabolic pathways, such as energy production, amino acid biosynthesis, and cell membrane components, NMR-based metabolomics can reveal precisely how an antimicrobial agent disrupts bacterial function.

In this context, the present study aims to evaluate the antibacterial potential of silver nanoparticles functionalized with hesperidin (AgNP@HSD) and hesperetin (AgNP@HST) against *X. axonopodis* pv. *citri*, integrating microbiological, metabolomic, and morphological approaches to better understand the mechanisms underlying AgNP antimicrobial activity.

## Methodology

### Synthesis of Silver Nanoparticles Functionalized with Hesperidin and Hesperitin

Biogenic silver nanoparticles were synthesized using 1 mmol L^–1^ AgNO_3_ as the silver ion source. As reducing agents, hesperetin 2 g L^–1^ (6.6 mmol L^–1^) and hesperidin 2 g L^–1^ (3.3 mmol L^–1^) were dissolved in 50 mL of 0.1 mmol L^–1^ NaOH solution. The AgNO_3_ solution was added dropwise to the reducing solution under constant magnetic stirring for 1 h and then protected from light for 72 h to form silver nanoparticles functionalized with hesperidin (AgNP@HSD) and hesperetin (AgNP@HST).

The nanoparticles were characterized by Dynamic Light Scattering (DLS) to determine their hydrodynamic diameter, size distribution, and surface charge. Measurements were performed at 25 °C on a Zetasizer Nano-ZS ZEN3600 instrument (Malvern Instruments, U.K.) equipped with a 4 mW He–Ne laser (633 nm) and a scattering angle of 173°. Each sample was measured in 15 acquisitions, and the results were reported as mean ± standard deviation from three independent measurements. Data analysis was conducted using Zetasizer software v7.11.

To confirm nanoparticle functionalization, Fourier Transform Infrared Spectroscopy (FTIR) was performed on an Agilent Cary 630 FTIR (Agilent Technologies) equipped with an attenuated total reflectance module.

### *X. axonopodis* pv. *citri* Culture and Antimicrobial Potential Evaluation

*X. axonopodis* pv. *citri* strain 306 (ibsbf 1594, São Paulo, Brazil) was cultured in SB medium containing sucrose (5 g L^–1^), yeast extract (5 g L^–1^), peptone (5 g L^–1^), and glutamic acid (1 g L^–1^). To determine the minimal inhibitory concentration (MIC), bacterial cultures were incubated in 96-well plates at 25 °C with constant shaking (200 rpm) for 24 h. The first column served as a positive control, and the second column as a negative control treated with 100 μg mL^–1^ streptomycin. Nanoparticle concentrations of 0.1, 0.2, 0.3, 0.75, 1.5, 3, 6, 12, 24, and 36 μg mL^–1^ were used to evaluate antibacterial activity. Bacterial growth was monitored by measuring absorbance at 600 nm (*A*_600_) using an Anthos Zenyth 200rt plate reader (Biochrom, U.K.). The MIC was defined as the lowest nanoparticle concentration that showed a statistically significant reduction in growth (*p* < 0.05) compared to the positive control.

For metabolic and morphological analysis, bacteria were incubated with 1.93 μg mL^–1^ of either AgNP@HSD or AgNP@HST for 24 h. Control cells were grown without nanoparticles. Cultures were centrifuged at 3500 rpm for 5 min at 4 °C, and pellets were resuspended in 20 mmol L^–1^ sodium phosphate buffer containing 150 mmol L^–1^ NaCl (pH 7.4). Cells were lysed using a QR500 ultrasound cell disruptor (Eco-Sonics, Brazil), and lysates were centrifuged at 14,000 rpm for 2 min at 4 °C. The resulting supernatants were vacuum-dried using a SpeedVac SPD1010–115 (Thermo Electron). For whole-cell analysis, intact bacteria were resuspended in buffer (20 mmol L^–1^ sodium phosphate, 150 mmol L^–1^ NaCl, 10% glycerol, pH 7.4) and stored at −80 °C until analysis.

### Metabolomic Analysis of *X. axonopodis* pv. *citri* Treated with Silver Nanoparticles

Metabolic profiling was conducted via ^1^H NMR spectroscopy using two approaches:

Bacterial lysates were subjected to liquid–liquid extraction with water: chloroform (1:1 v/v). The nonpolar phase was vacuum-dried and resuspended in CDCl_3_ for NMR analysis. The polar fraction was similarly processed and resuspended in D_2_O. Spectra were acquired on a Bruker Avance NEO 600 MHz spectrometer (Bruker) equipped with an iProbe SmartProbe using Triple Broadband Observe (TBO) and Broadband Frequency (BBF) channels and a 24-position autosampler.

For intact cell analysis, bacteria were diluted in D_2_O to reach a 10% D_2_O final concentration, and 12 μL were loaded into a 4 mm High Resolution – Magic Angle Spinning (HR-MAS) rotor insert. Spectra were acquired using a Bruker Avance III 400 MHz spectrometer with an HR-MAS probe, spinning at 5000 Hz.

All NMR experiments used the Carr–Purcell–Meiboom–Gill (CPMG) pulse sequence with T_2_ filtering and presaturation (cpmgpr1d). Each sample was analyzed with 128 scans for liquid-phase NMR and 256 scans for HR-MAS. Spectra were processed in TopSpin 4.5.01 with manual phase and baseline correction. Chemical shifts were referenced to TSP (δ 0.00).

### Chemometrics and Statistical Analysis

Chemometric analysis was performed using MetaboAnalyst 6.0.^38^ Spectra were aligned using TSP (δ 0.00) and binned at 0.06 ppm intervals. Before multivariate analysis, data were normalized to the median and autoscaled. Partial Least-Squares-Discriminant Analysis (PLS-DA) was used to evaluate group separation. For univariate analysis, one-way Analysis of Variance (ANOVA) was conducted, followed by Tukey’s post hoc test to correct for multiple comparisons when identifying metabolites with statistically significant differences (*p* < 0.05). Metabolite assignment was based on chemical shifts, coupling constants, and peak multiplicities, and validated with reference databases.

### Scanning Electron Microscopy of *X. axonopodis* pv. *citri*

For Scanning Electron Microscopy (SEM) analysis, bacterial cultures were first centrifuged at 3500 rpm for 5 min at room temperature to collect the cells, and the resulting pellets were washed three times with sodium phosphate buffer 100 mmol L^–1^. Fixation was carried out by adding 0.25% glutaraldehyde prepared in 100 mmol L^–1^ sodium phosphate buffer (pH 7.2), followed by incubation at room temperature for 30 min and an additional overnight incubation at 4 °C. After fixation, the samples were washed three more times with 100 mmol L^–1^ sodium phosphate buffer and centrifuged at 3500 rpm for 5 min at room temperature to collect the pellet. The samples were then dehydrated using a graded ethanol series (30%, 50%, 70%, 80%, 90%, and 100%), with each step lasting 10 min, followed by an additional incubation in 100% ethanol for 1 h. Finally, the bacterial samples were mounted on SEM stubs using double-sided adhesive carbon tape for imaging. The micrographics were taken using a Quanta FEG 250 (Thermo Fisher).

## Results and Discussion

### AgNP@HSD and AgNP@HST Synthesis and Characterization

The size and surface charge of the silver nanoparticles were characterized by dynamic light scattering (DLS). The AgNP@HSD particles exhibited a hydrodynamic diameter of 67 ± 11 nm and a ζ-potential of −34 ± 6 mV. In contrast, AgNP@HST showed a slightly smaller hydrodynamic diameter of 63 ± 8 nm and a ζ-potential of −43 ± 9 mV. The DLS results for both nanoparticles are presented in Figure 1.

**Figure 1:**
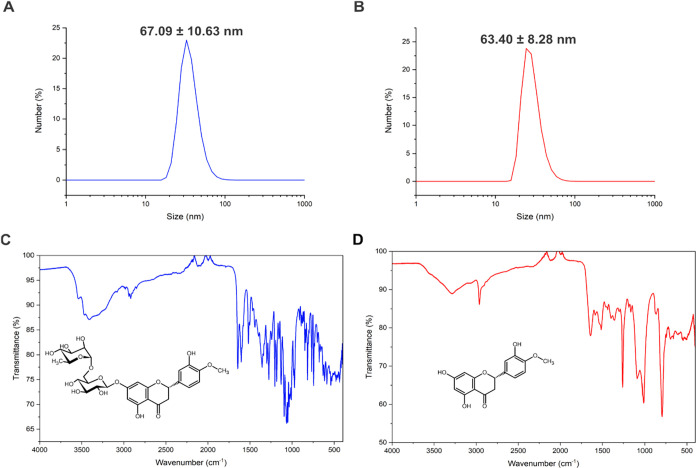
DLS size distribution diagram of (A) AgNP@HSD showing an average size of 67.09 ± 10.63 nm and (B) AgNP@HST showing an average size of 63.40 ± 8.48 nm. FITR spectra of (C) AgNP@HSD and (D) AgNP@HST.

FTIR spectra of both nanoparticles are shown in Figure 1. The spectrum of AgNP@HSD displays characteristic bands of hesperidin,^39–41^ including a broad absorption at 3418 cm^–1^ corresponding to O–H stretching, and bands at 2920 cm^–1^ and 1519 cm^–1^ attributed to C–C and C=C stretching, respectively. A prominent band at 1600 cm^–1^ indicates the presence of C=O stretching, while additional bands at 1260 cm^–1^ and 1050 cm^–1^ are associated with C–O stretching in ether linkages, supporting successful conjugation with hesperidin.

The infrared spectrum of AgNP@HST shows the characteristic vibrational bands of hesperetin.^39,42,43^ A broad band at 3300 cm^–1^ corresponds to O–H stretching, while the signal at 2917 cm^–1^ is attributed to C–C stretching. The presence of aromatic rings is confirmed by the band at 1462 cm^–1^. Additionally, a strong absorption at 1648 cm^–1^ indicates C=O stretching, and the band at 1298 cm^–1^ is consistent with C–O stretching in ether groups.

### Functionalized Silver Nanoparticles: Antibacterial Activity Potential

The antibacterial activity of silver nanoparticles functionalized with hesperidin and hesperetin was evaluated against *X. axonopodis* pv. *citri* using absorbance at 600 nm (*A*_600_) as a measure of bacterial growth. The results are presented in Figure 2. Both nanoparticle treatments significantly inhibited bacterial growth in a concentration-dependent manner. AgNP@HST exhibited higher antibacterial activity than AgNP@HSD, with a minimal inhibitory concentration of 12 μg mL^–1^, compared to 24 μg mL^–1^ for AgNP@HSD (*N* = 7). The key physicochemical properties and antimicrobial activities of the synthesized nanoparticles are summarized in Table 1.

**Figure 2:**
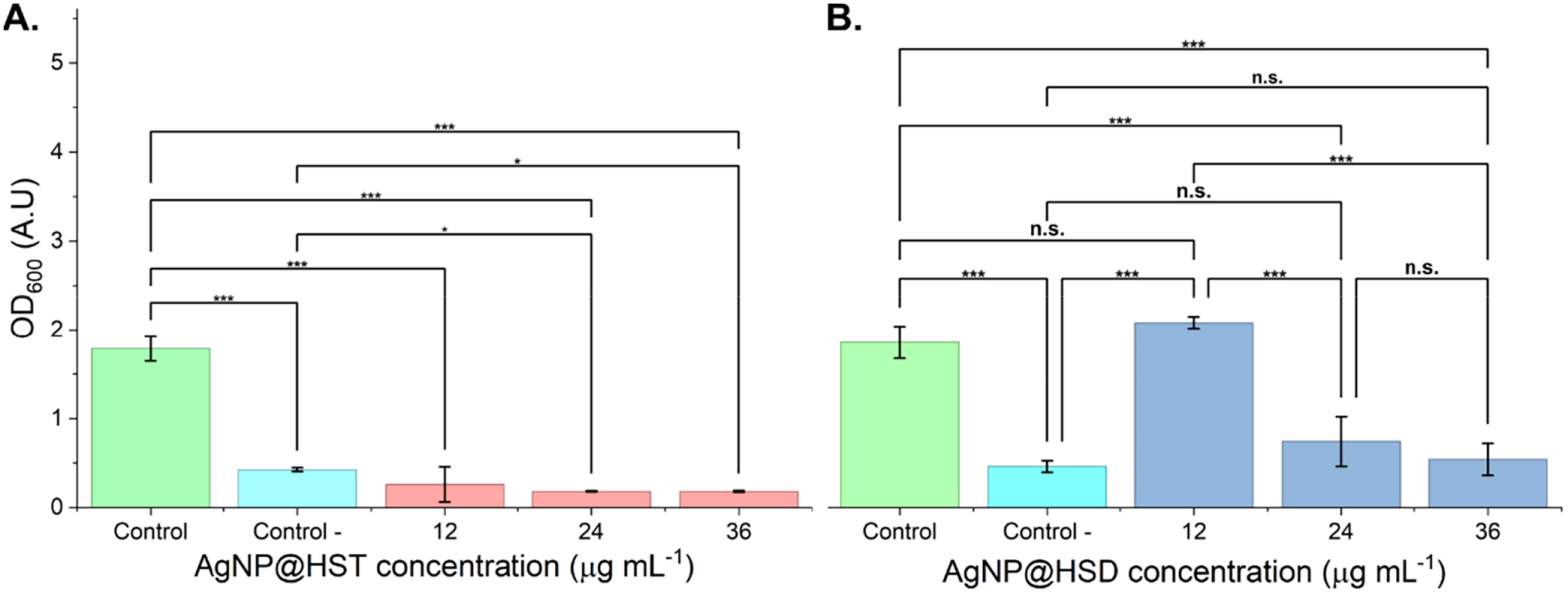
Antibacterial activity in *X. axonopodis* pv. *citri* of silver nanoparticles functionalized with (A) Hesperitin - AgNP@HST and (B) Hesperidin - AgNP@HSD. Absence of antibiotic or nanoparticle was used as positive control (green), streptomycin 100 μg mL^–1^ was used as negative control (cyan). *p < 0.05, ** *p* < 0.01, ***p < 0.001, n.s. = Not significant, *N* = 7.

**Table 1:** Physicochemical Properties and Minimum Inhibitory Concentrations of Flavonoid-Functionalized Silver Nanoparticles against *X. axonopodis* pv. *citri*

Property	AgNP@HSD	AgNP@HST
Hydrodynamic diameter	67 ± 11 nm	63 ± 8 nm
ζ-potential	–34 ± 6 mV	–43 ± 9 mV
key FITR peaks	3418 cm^–1^ (O–H), 1600 cm^–1^ (C=O), 1050 cm^–1^ (CO glycosidic)	3300 cm^–1^ (O–H), 1648 cm^–1^ (C=O), 1298 cm^–1^ (CO ether)
MIC	24 μg mL^–1^	12 μg mL^–1^

### Metabolomic Profiles

The nuclear magnetic ressonance-based (NMR-based) metabolomic profiles of *X. axonopodis* pv. *citri* were analyzed using PLS-DA. The polar and nonpolar extracts were examined by ^1^H NMR spectroscopy, while the intact whole cells were analyzed using HR-MAS ^1^H NMR. Figure 3 presents the PLS-DA scores plot of the polar phase (*N* = 3 for control treatment, *N* = 4 for other treatments), revealing three well-defined clusters that clearly differentiate the control group from the bacteria treated with AgNP@HSD and AgNP@HST. The separation is primarily driven by variations in the relative concentrations of *cis*-aconitate, ATP, nicotinate, and niacinamide.

**Figure 3:**
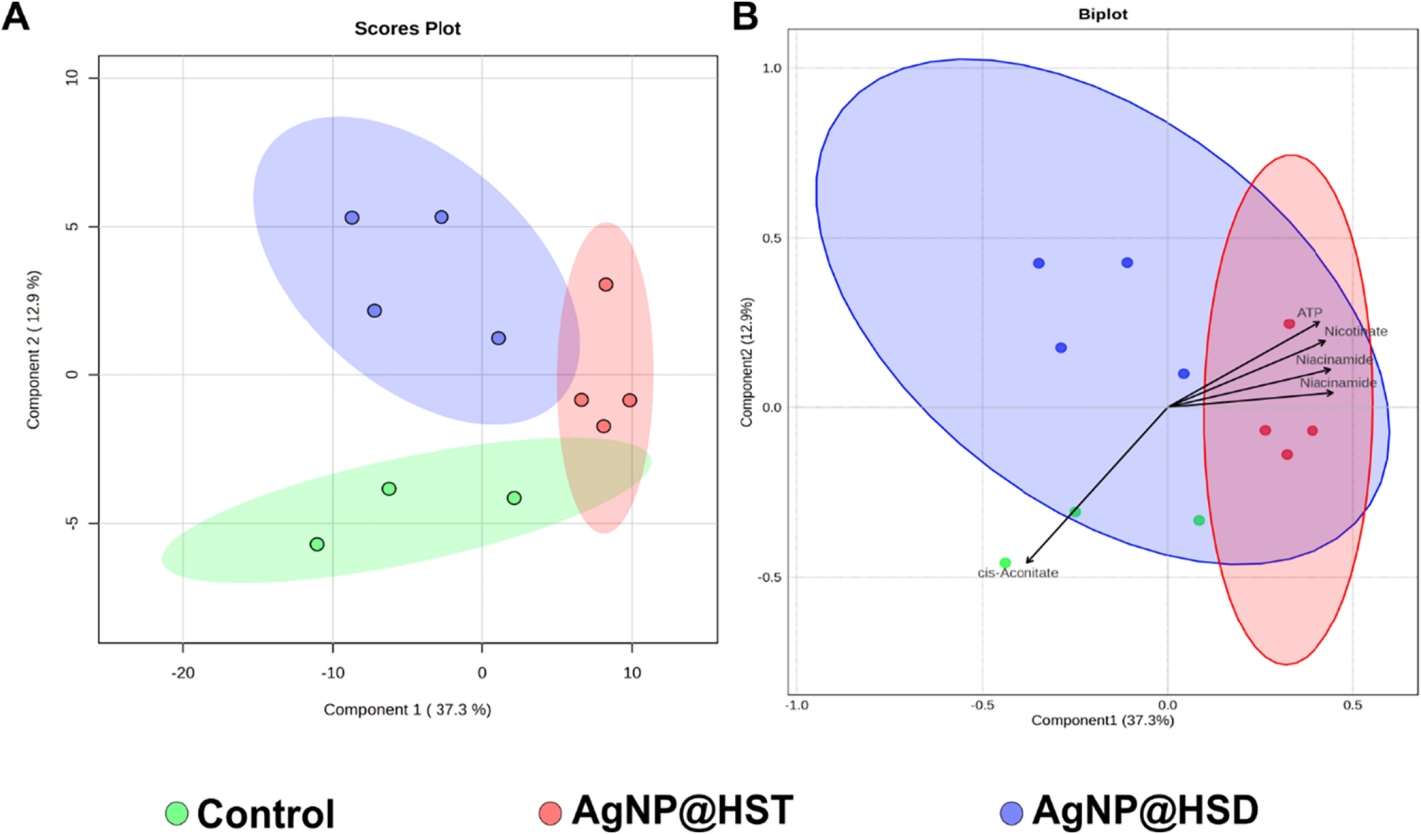
(A) Scores plot of PLS-DA analysis of polar phase showing the differences between the control (green), AgNP@HSD (blue), and AgNP@HST (red) groups, with principal component 1 on the *x*-axis explaining 37.3% of the variance and principal component 2 on the *y*-axis explaining 12.9%. Model performance: *Q* ^2^ = 0.23, *R* ^2^ = 0.58, accuracy = 0.67. (B) Combined scores and loadings plot showing the principal metabolites’ contributions to the model separation (arrows). The arrows’ lengths and directions indicate the influence of the respective metabolite separation between the groups (*N* = 3 for the control group, *N* = 4 for the AgNP@HSD and AgNP@HST treated groups).

Ten metabolites exhibited statistically significant differences among the groups: arginine (δ 1.62), formate (δ 8.44), glutamine (δ 2.10), lysine (δ 1.43), methionine (δ 2.16), *N*-acetyl-glutamine (δ 2.22), phosphoserine (δ 4.19), proline (δ 2.04), threonine (δ 3.59), and valine (δ 2.28). Figure 4 displays the variation in relative intensity for each metabolite across the treatment groups. Figure 5 shows the PLS-DA of the nonpolar phase (*N* = 4), highlighting a clear separation between the treatments. The biplot indicates that the group separation is mainly influenced by changes in the peaks corresponding to methyl groups at the terminal positions of fatty acid chains (δ 1.02) and methyl substituents in triacylglyceride chains (δ 1.92). Peaks from methylene groups (CH_2_) in fatty acid chains (δ 1.38, δ 1.50) and those adjacent to carboxyl groups (δ 2.16, δ 2.40) also contribute to the separation. All these peaks displayed significantly increased relative intensities (*p* < 0.05, Figure 6) in the nanoparticle-treated groups, especially in the AgNP@HSD group, compared to the control.

**Figure 4:**
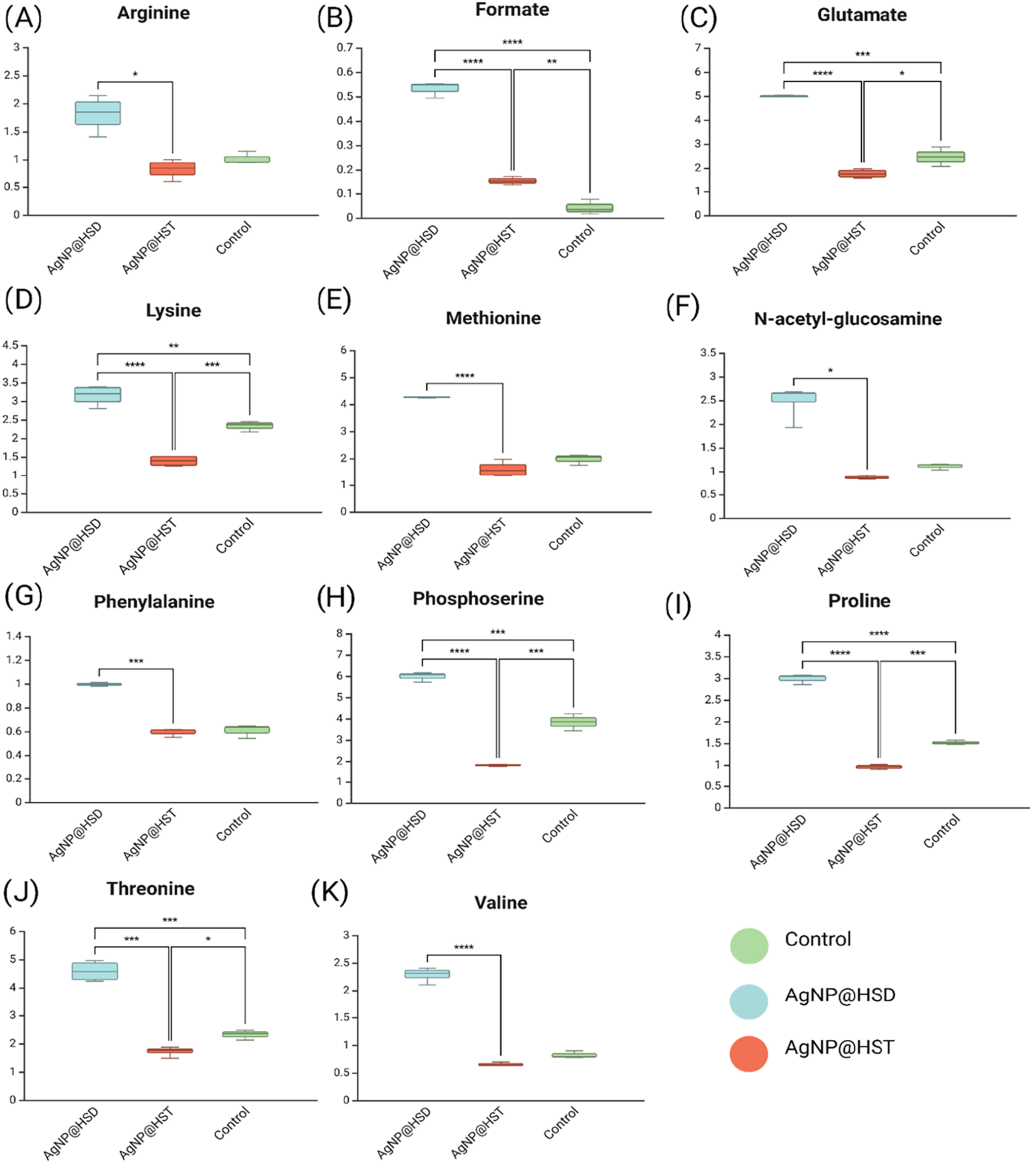
Boxplots with statistically significantly different metabolites between the AgNP@HST, AgNP@HSD treated, and control groups in the polar phase. Bar plots represent mean ± standard deviation of metabolite levels detected by ^1^H NMR spectroscopy (A) Arginine, (B) Formate, (C) Glutamate, (D) Lysine, (E) Methionine, (F) *N*-acetyl-glucosamine, (G) Phenylalalnine, (H) Phosphoserine, (I) Proline, (J) Threonine, (K) Valine. *p < 0.05, ** *p* < 0.01, ***p < 0.001. *N* = 4 for *X. axonopodis* pv. *citri* treated with nanoparticle, *N* = 3 for the control group.

**Figure 5:**
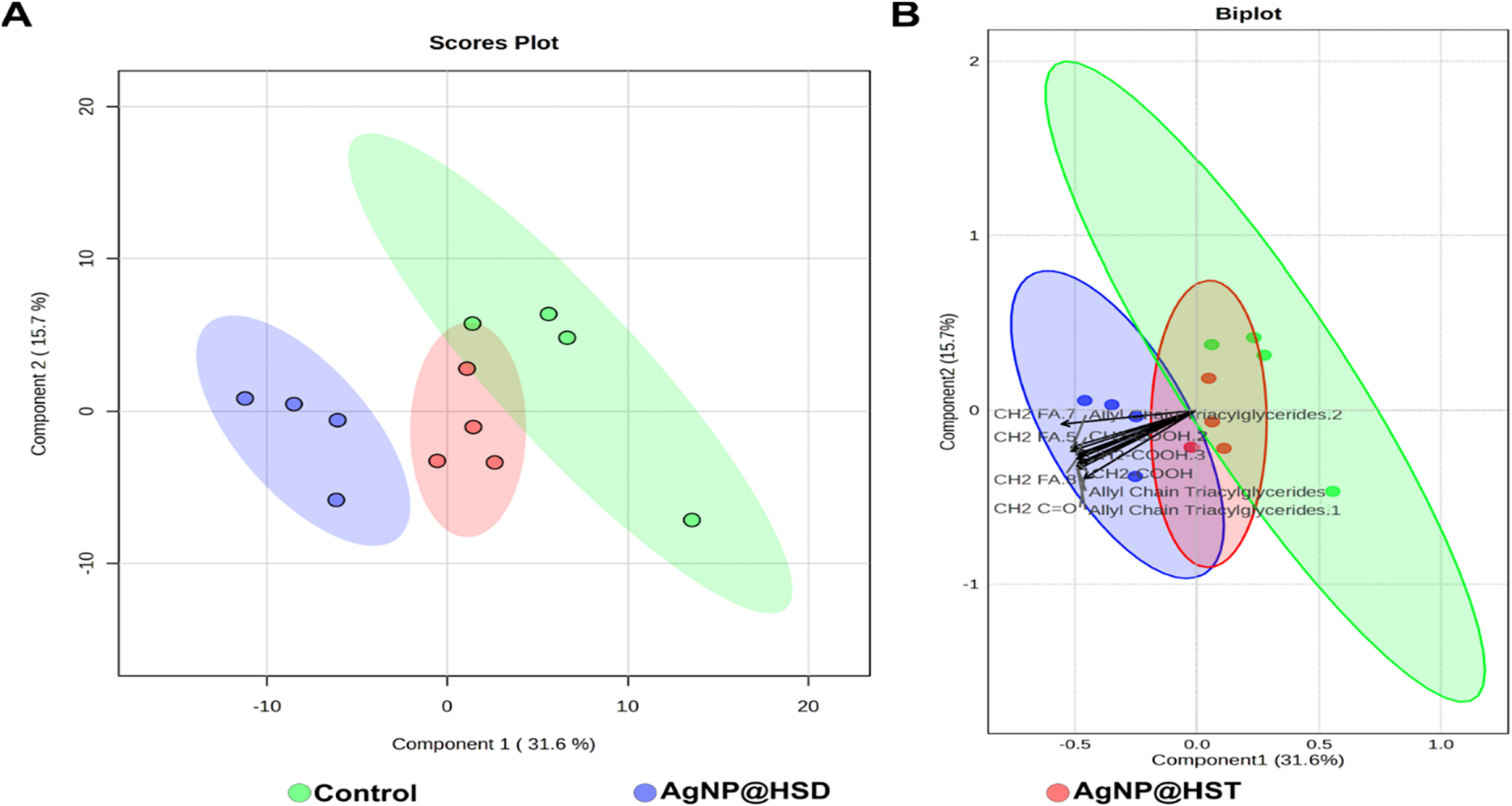
(A) Scores plot of PLS-DA analysis of nonpolar phase showing the differences between the control (green), AgNP@HSD (blue), and AgNP@HST (red) groups, with principal component 1 on the *x*-axis explaining 31.6% of the variance and principal component 2 on the *y*-axis explaining 15.7%. Model performance: *Q* ^2^ = 0.44, *R* ^2^ = 0.71, accuracy = 0.6. (B) Combined scores and loadings plot showing the principal metabolites’ contributions to the model separation (arrows). The arrows’ lengths and directions indicate the influence of the respective metabolite separation between the groups (*N* = 4).

**Figure 6:**
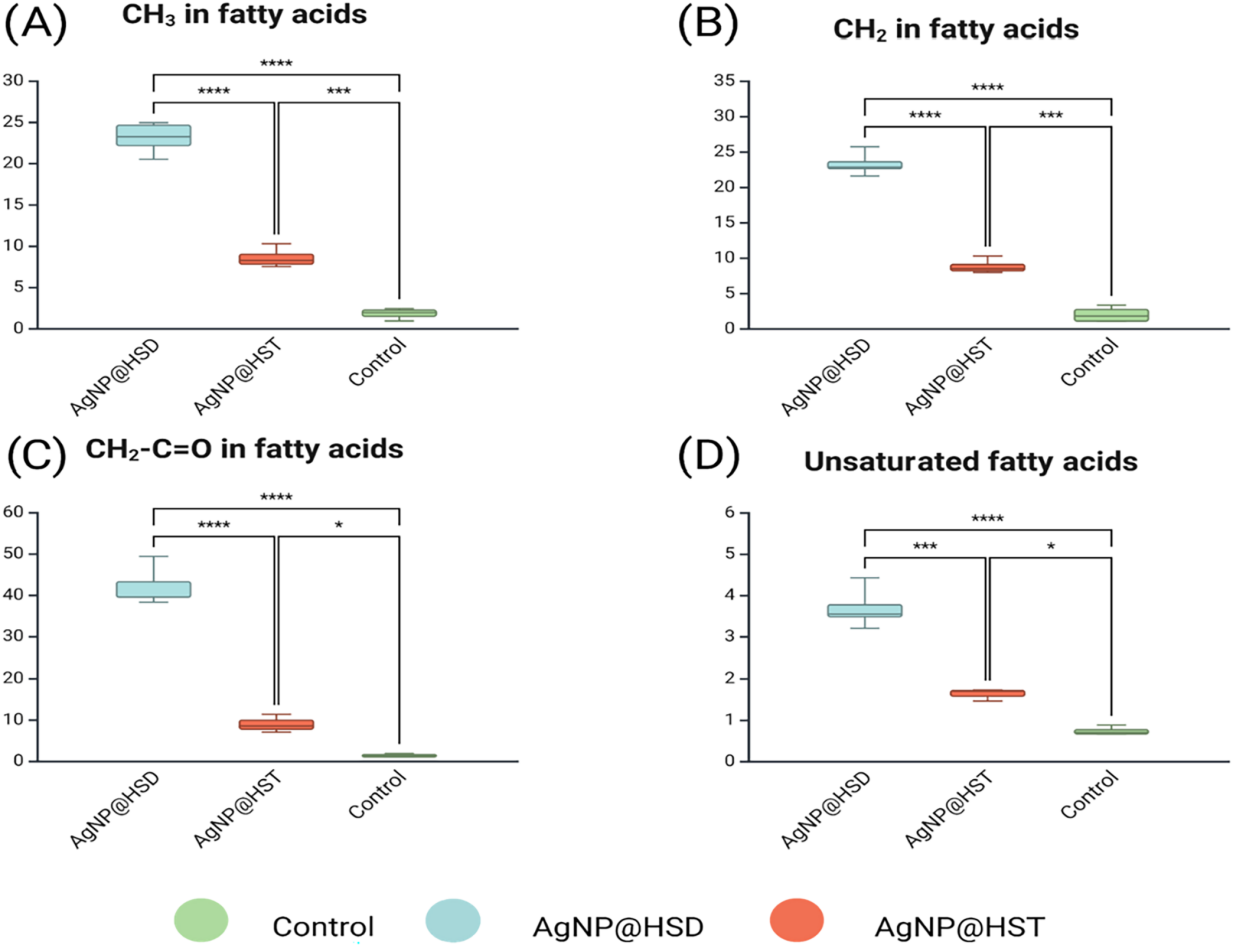
Boxplots with statistically significantly different metabolites between the AgNP@HST, AgNP@HSD treated, and control groups in the nonpolar phase. Bar plots represent mean ± standard deviation of metabolite levels detected by ^1^H NMR spectroscopy (A) CH_3_ in fatty acids, (B) CH_2_ in fatty acids, (C) CH_2_ linked to carboxy group in fatty acids (D) Unsaturated fatty acids. *p < 0.05, ** *p* < 0.01, ***p < 0.001. *N* = 4 for all *X. axonopodis* pv. *citri* treated and control groups.

The chemometric analysis of whole cells analyzed by HR-MAS ^1^H NMR (Figure 7) showed a clear separation between the control and the AgNP@HST group, whereas the AgNP@HSD and control groups did not separate distinctly (*N* = 3 for AgNP@HSD treatment, **N** = 4 for other treatments). The associated biplot suggests that this separation is influenced by changes in the relative intensities of peaks corresponding to glyceryl phosphate groups (δ 5.26), triacylglycerides (δ 5.15), acyl chains in fatty acids (δ 5.45), isoleucine (δ 1.25), glutamine (δ 2.45), niacinamide (δ 8.74), nicotinate (δ 8.62), and NAD­(P)H (δ 8.80, δ 8.86). Additionally, ATP (δ 8.26, δ 8.56), formate (δ 8.44), and UDP-*N*-acetyl-glucosamine (δ 7.96, δ 8.38) also showed statistically significant differences in peak intensities among the groups. These differences are illustrated in the bar plots shown in Figure 8.

**Figure 7:**
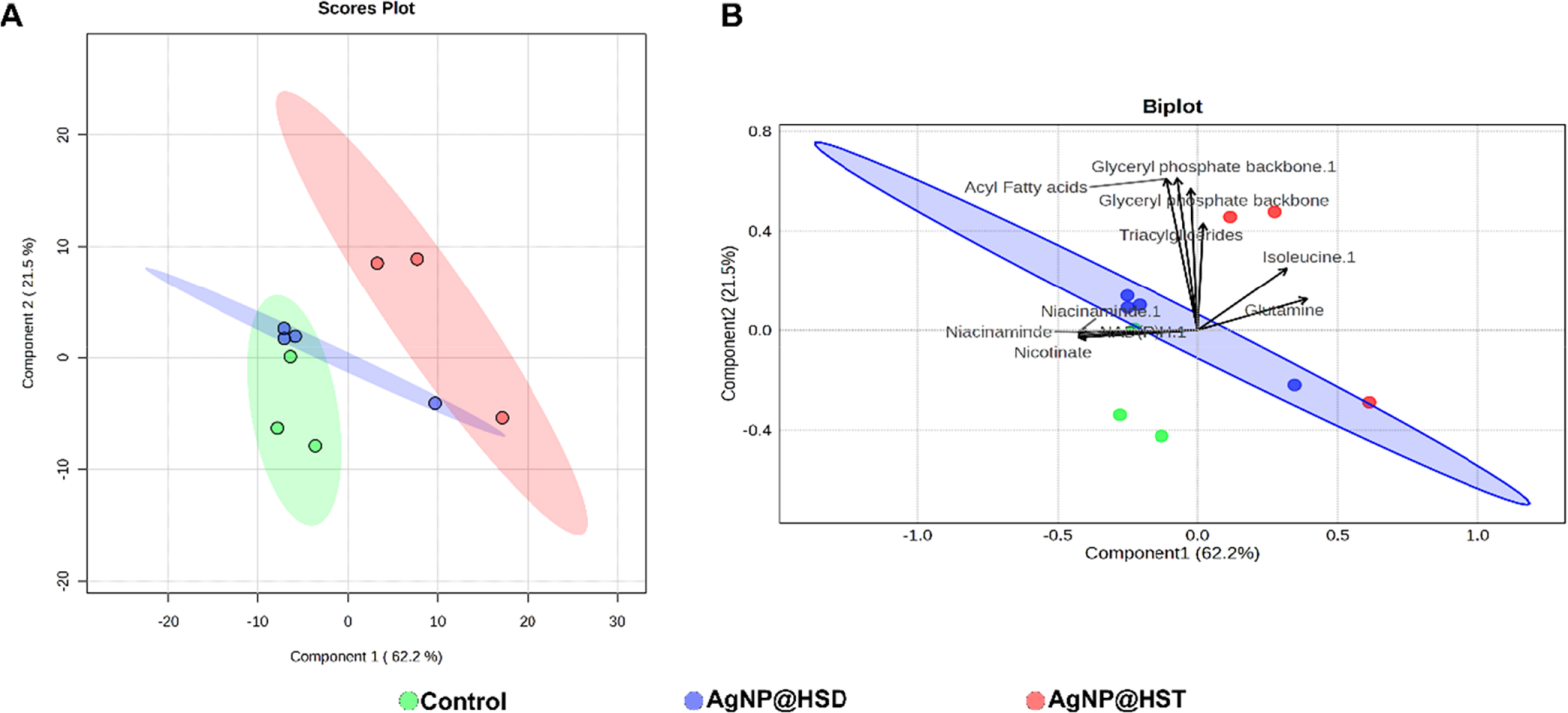
(A) Scores plot of PLS-DA analysis of bacteria whole cell showing the differences between the control (green), AgNP@HSD (blue), and AgNP@HST (red) groups, with principal component 1 on the *x*-axis explaining 62.2% of the variance and principal component 2 on the *y*-axis explaining 21.5%. Model performance: *Q* ^2^ = 0.57, *R* ^2^ = 0.86, accuracy = 0.367. (B) Combined scores and loadings plot showing the principal metabolites’ contributions to the model separation (arrows). The arrows’ lengths and directions indicate the influence of the respective metabolite separation between the groups (*N* = 3 for AgNP@HSD treatment, *N* = 4 for Control and AgNP@HST groups).

**Figure 8:**
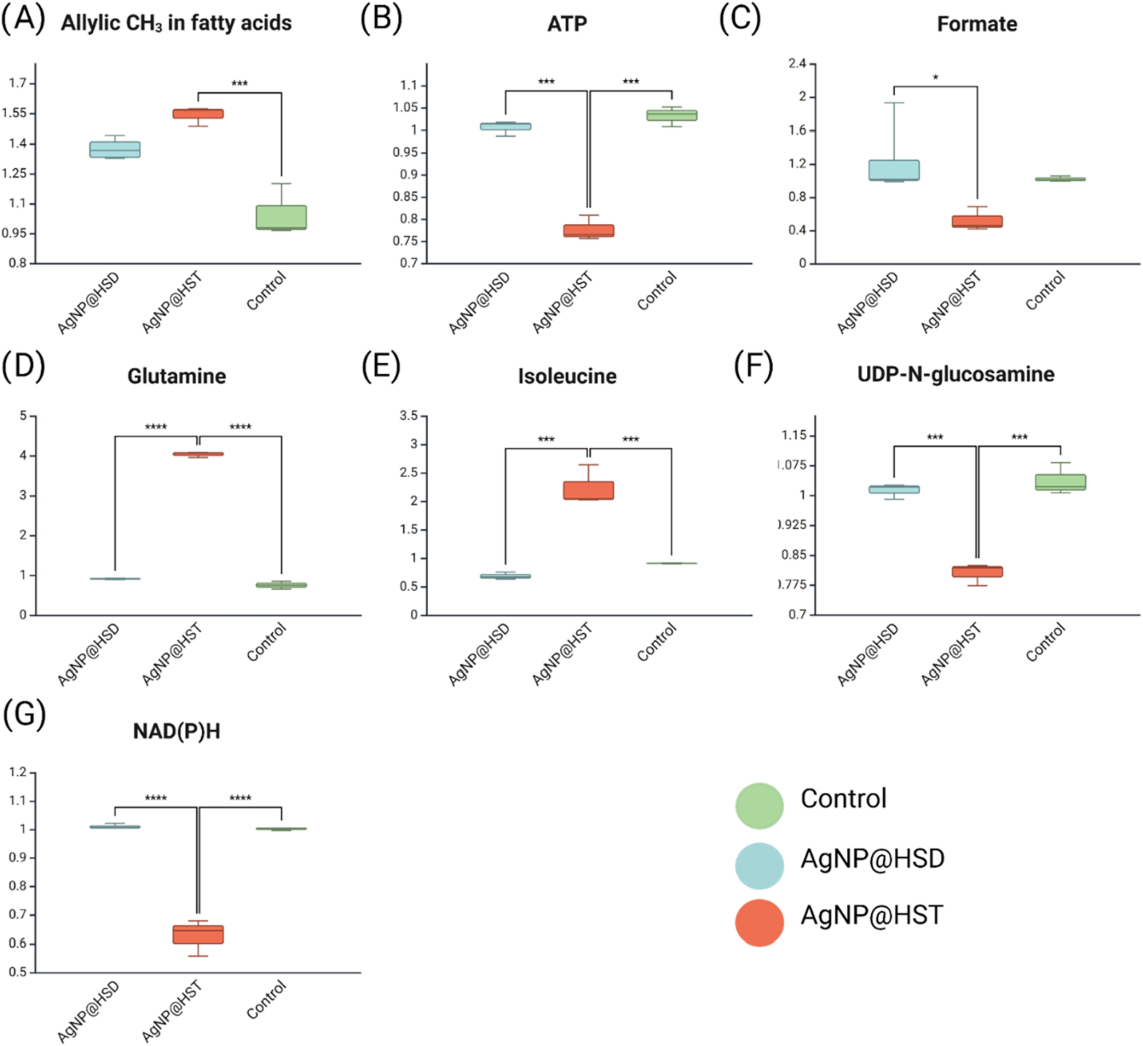
Boxplots with statistically significantly different metabolites between the AgNP@HST, AgNP@HSD treated, and control groups in whole cell analysis. Bar plots represent mean ± standard deviation of metabolite levels detected by ^1^H NMR spectroscopy (A) Allylic CH_3_ in fatty acids, (B) ATP, (C) Formate, (D) Glutamine, (E) Isoleucine, (F) UDP-*N*-glucosamine, (G) NAD­(P)­H. *p < 0.05, ** *p* < 0.01, ***p < 0.001. *N* = 4 for *X. axonopodis* pv. *citri* treated with AgNP@HSD and control groups, *N* = 3 for bacteria treated with AgNP@HST.

The synthesized silver nanoparticles had a size of 67 ± 11 nm for AgNP@HSD, AgNP@HST showed a marginally different hydrodynamic diameter of 63 ± 8 nm in DLS measurements, which is accordingly with the literature, usually reports silver nanoparticles in range of sizes between 1 to 100 nm^44–46^ and, the range of size smaller than 90 nm shows a higher antibacterial activity.^41^ The ζ-potential value of −34 ± 6 mV for AgNP@HSD indicates relatively stable nanoparticles, considering that values lower than −35 mV and higher than 42 mV indicate electrostatically stable particles.^47–49^ On the other hand, AgNP@HST showed a lower ζ-potential of −43 ± 9 mV, indicating electrostatically more stable nanoparticles.

The FTIR characterization confirmed the successful functionalization of silver nanoparticles with hesperetin and hesperidin by identifying their characteristic vibrational bands.

In the AgNP@HST FTIR spectrum, bands around ∼3300 cm^–1^ and ∼2917 cm^–1^ correspond to O–H and C–H stretching vibrations, respectively. A sharp peak at 1648 cm^–1^ indicates C=O stretching, while the band at 1462 cm^–1^ is associated with aromatic ring vibrations. The C–O stretching vibration, characteristic of ether groups, appears at 1298 cm^–1^.

In contrast, the FTIR spectrum of AgNP@HSD displays additional bands around 1260 cm^–1^ and 1050 cm^–1^, which are characteristic of C–O stretching in glycosidic bonds. These bands, along with the flavonoid-associated signals mentioned above, confirm the presence of hesperidin on the nanoparticle surface.

Overall, the presence and preservation of these functional groups support the successful surface functionalization of silver nanoparticles with each flavonoid. Notably, the glycosidic C–O bands observed in AgNP@HSD but absent in AgNP@HST highlight the structural distinction between hesperidin (a glycosylated flavonoid) and hesperetin (its aglycone form). These structural differences may significantly influence the nanoparticles’ biological activity and their interaction with bacterial membranes.

The MIC assay demonstrated that AgNP@HST exhibited higher antibacterial activity against *X. axonopodis* pv. *citri* compared to AgNP@HSD, with a MIC of 12 μg mL^–1^ versus 24 μg mL^–1^, respectively. Notably, both functionalized nanoparticles showed antibacterial effects comparable to streptomycin, which was used at a substantially higher concentration (100 μg mL^–1^). Among the treatments, AgNP@HST was the most effective, resulting in significantly lower bacterial growth (*A*_600_) than even the negative control group treated with streptomycin. These findings highlight the strong antibacterial potential of silver nanoparticles functionalized with flavonoids, particularly AgNP@HST, and support their further investigation as promising alternative antimicrobial agents.

While the potent antimicrobial activity of the biofunctionalized nanoparticles is evident, distinguishing the individual contributions of the silver core and the flavonoid coating is crucial. Although AgNPs, hesperidin, and hesperetin each possess intrinsic antibacterial properties, the literature strongly suggests that biofunctionalization enhances efficacy.^13^ For instance, chemically synthesized AgNPs often exhibit MICs exceeding 500 μg mL^–1^ against similar bacteria,^50–52^ while hesperetin and hesperidin typically show MICs around 125 μg mL^–1^ and 1000 μg mL^–1^, respectively.^23^ In contrast, the biofunctionalized formulations in this study achieved MICs of 12 μg mL^–1^ for AgNP@HST and 24 μg mL^–1^ for AgNP@HSD. These values are significantly lower than those reported for the isolated components, suggesting a potent synergistic effect. Despite this strong indication of synergy, a definitive confirmation of this effect would require additional experiments using each component as a separate control.

The metabolomic profiles obtained through ^1^H NMR analyses reveal distinct biochemical responses of *X. axonopodis* pv. *citri* to silver nanoparticles functionalized with hesperidin and hesperetin. These differences reflect how each flavonoid modulates bacterial metabolism, likely due to their structural variations and resulting differences in biological activity. Previous studies have shown that hesperetin, exhibits greater antibacterial activity, which is attributed to the absence of the rutinose moiety in its structure.^23,24^ However, silver nanoparticles functionalized with hesperidin have also demonstrated promising biological properties, not only as antioxidant and antitumoral agents,^53^ but also with antibacterial potential,^54^ suggesting that nanoparticle formulation may enhance or alter the native activity of the flavonoid.

The analysis of the polar fractions revealed that treatment with AgNP@HSD induced a broader metabolic response compared to AgNP@HST. Specifically, hesperidin-coated AgNPs led to significant increases of several amino acids, including formate, glutamine, lysine, methionine, phenylalanine, threonine, proline, and valine. In particular, the increased level of phosphoserine suggests a potential alteration in the tetrahydrofolate (THF) pathway. This metabolic route is a promising target for next-generation antibiotics^55–57^ and is closely linked to the biosynthesis of different amino acids and cellular energy metabolism, as shown in Figure 9. Further evidence of bioenergetics changes is the variation in *cis*-aconitate, an intermediate of the Krebs cycle, and NADH precursors, such as niacinamide and nicotinamide. Notably, NAD­(P)­H itself also exhibited variations in the whole-cell analysis.

**Figure 9:**
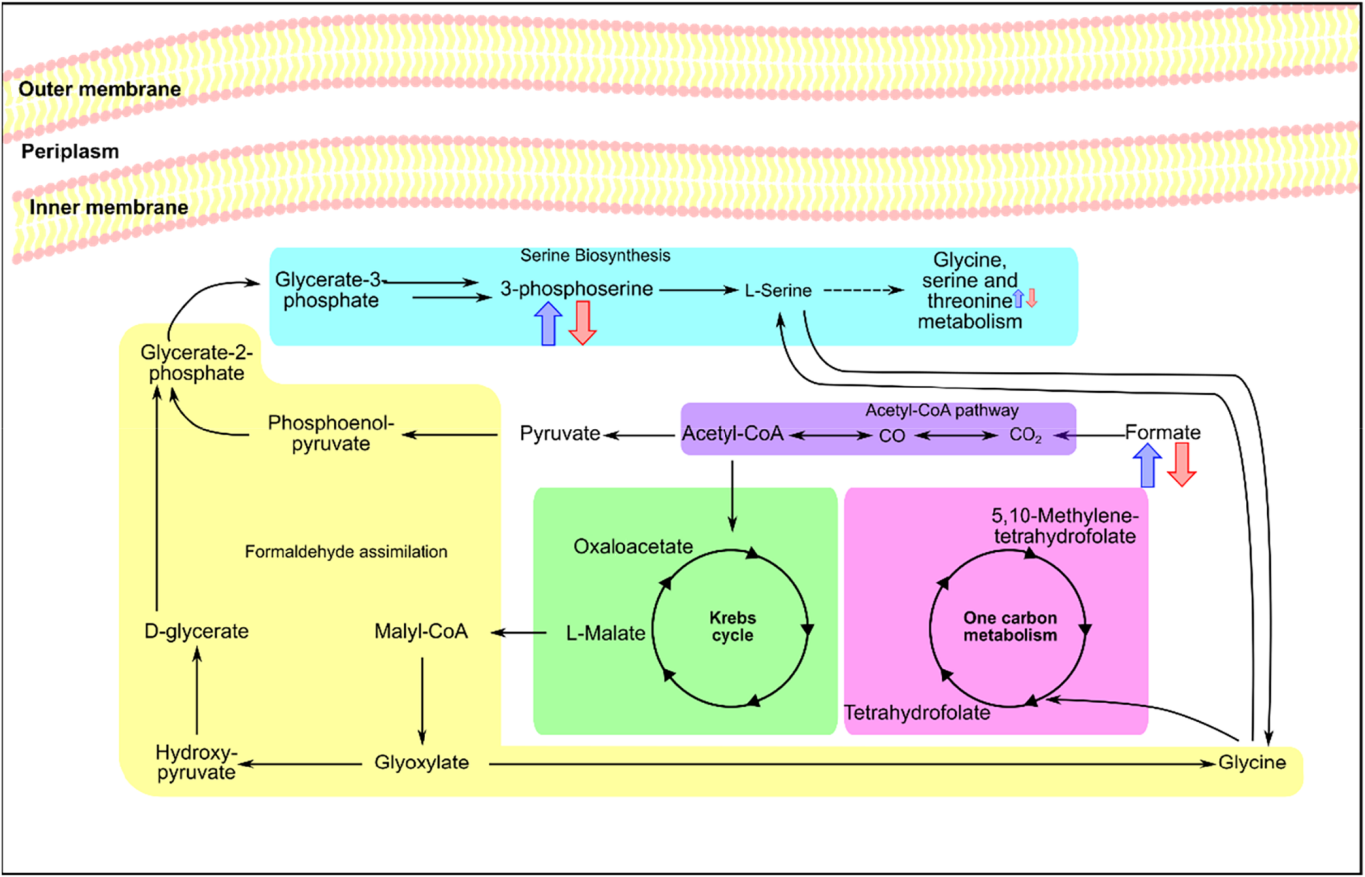
Schematic representation of key metabolic pathways altered in *X. axonopodis* pv. *citri* after treatment with AgNP@HSD and AgNP@HST. Metabolites showing relative intensity changes in ^1^H NMR are marked in blue arrows for bacteria treated with AgNP@HSD, and red arrows for bacteria treated with AgNP@HST. Up arrows indicate an increase in relative intensity and down arrows indicate a decrease.

The nonpolar phase results revealed an increase in the signal intensity of methyl groups at the terminal positions of fatty acid chains in both nanoparticle-treated groups, suggesting possible alterations in membrane lipid lipid-associated signals, including methyl substituents in triacylglyceride chains, methylene (CH_2_) groups in fatty acids, and unsaturated fatty acids. These findings suggest AgNP@HSD prompts more significant membrane lipid remodeling, potentially as a compensatory stress response. It is important to note, however, that this interpretation is constrained by the performance of our chemometric model.

The analysis yielded a *Q* ^2^ of 0.44 and an *R* ^2^ of 0.71, which indicates moderate predictive power. This limitation means the model may be unable to resolve all biologically relevant differences, particularly subtle metabolic effects. For instance, the minor lipid alterations suspected in the AgNP@HST group may have been obscured by factors such as overlapping NMR signals, a small sample size, and inherent biological variability. Finally, we acknowledge a limitation regarding the unequal number of biological replicates across the experimental groups in metabolomic analyses. Specifically, the control group in the polar phase analysis (Figure 3) and the AgNP@HST group in the whole-cell analysis (Figure 7) included three replicates (*N* = 3), while other groups included four (*N* = 4). While we recognize that a balanced design is statistically preferable, the PLS-DA models successfully discriminated between the groups with high validation metrics (*e.g.*, *R* ^2^ = 0.86 for whole-cell analysis), suggesting that the metabolic impact of the nanoparticles was robust enough to overcome the variance introduced by the sampling imbalance. Nevertheless, future investigations will ensure a strictly uniform sample size to maximize statistical rigor.

AgNPs have been widely reported as potent inducers of oxidative stress, a property that has been increasingly explored to combat drug-resistant bacteria.^58^ Evidence suggests that the generation of reactive oxygen species from the nanoparticle surface can lead to increased membrane permeability and oxidative damage to bacterial membranes.^59^ Several studies highlight the antibacterial activity of AgNPs, showing a generally higher efficacy against Gram-negative bacteria compared to Gram-positive strains, with membrane disruption frequently identified as a key mechanism of action.^60–63^ Scanning electron microscopy SEM was used to examine morphological changes in the *X. axonopodis* pv. *citri* cell membrane, as shown in Figure 10. Clear differences in membrane integrity were observed between the control and nanoparticle-treated groups. While the control cells exhibited intact and well-defined membranes, signs of membrane disruption were evident in both AgNP-treated groups. Notably, membrane degradation appeared more pronounced in the AgNP@HSD group compared to the AgNP@HST group, suggesting a stronger impact of hesperidin-functionalized nanoparticles on membrane integrity.

**Figure 10:**
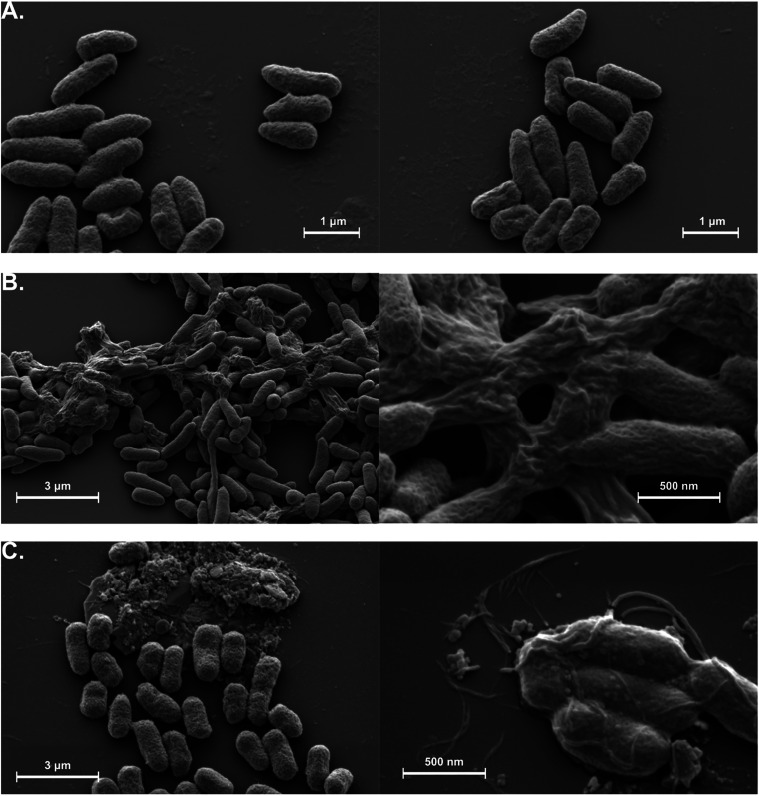
Scanning electron microscopy images of *X. axonopodis* pv. *citri* cells after treatment with silver nanoparticles. (A) Untreated control cells, (B) bacteria treated with AgNP@HSD, (C) bacteria treated with AgNP@HST. All images were acquired with different magnifications to highlight morphological alteration at single-cell and population levels.

In the intact-cell HR-MAS ^1^H NMR analysis, several metabolic trends previously observed in solution-state NMR were confirmed. Peaks corresponding to allylic CH_3_ groups in fatty acids, glutamine, and isoleucine showed increased relative intensities, while UDP-*N*-acetylglucosamine, ATP, formate, and NAD­(P)H levels decreased. These changes likely reflect alterations in energy metabolism and cell wall biosynthesis, possibly as part of the bacterial adaptive response to oxidative stress and membrane damage induced by silver nanoparticles. Particularly relevant observations were the decreases in NAD­(P)H and ATP levels in the AgNP@HST-treated group. These suggest a disruption of redox homeostasis and impaired energy production caused by the hesperetin-functionalized nanoparticles. NAD­(P)H is a key cofactor involved in protecting cells against oxidative damage. In certain bacteria, the conversion of NADH to NAD­(P)H serves as a regulatory mechanism to modulate the oxidative stress response.^64,65^ In *Escherichia coli*, elevated NAD­(P)­H levels are associated with the repair and function of redox-active enzymes such as flavodoxin, flavodoxin/ferredoxin-NAD­(P)^+^ reductase, and OxyR-dependent peroxidases and reductases—all of which rely on NAD­(P)H as a reducing agent.^66^ Similarly, in *Pseudomonas putida*, a bacterium adapted to polluted environments, NAD­(P)H plays a central role in oxidative stress resistance by maintaining glutathione-dependent detoxification pathways.^66^ The reduced NAD­(P)H levels observed in the AgNP@HST group, compared to AgNP@HSD and control, may indicate a greater oxidative stress burden triggered by hesperetin-functionalized nanoparticles. Alternatively, it could reflect a distinct metabolic adaptation—such as a lipid-centered stress response—induced specifically by the hesperidin-based formulation. However, further multiomics investigations are required to elucidate the precise metabolic and regulatory effects of these two nanoparticle formulations in *X. axonopodis* pv. *citri*.

Collectively, these results suggest that AgNP@HSD induces a more global metabolic and structural stress response, possibly involving membrane remodeling and amino acid metabolism as bacterial defense strategies. Conversely, AgNP@HST appears to cause more targeted oxidative damage, as evidenced by NAD­(P)H depletion and distinct metabolic shifts. These differences are likely linked to the higher intrinsic bioactivity of hesperetin, the aglycone form of hesperidin, which is known to exhibit stronger antibacterial properties.

The distinct bioactive properties of each molecule can also explain the differences in MIC values. AgNP@HST appears to cause a more targeted metabolic disruption, inflicting severe damage that collapses the cell’s bioenergetics and leaves little room for adaptation. In contrast, the bacteria treated with AgNP@HSD display a broader range of metabolic changes, including lipid membrane remodeling and oxidative stress responses. These changes may represent a less effective adaptation strategy that ultimately also leads to cell death. The *in vitro* results are highly promising, nevertheless, the transition to field applications requires careful consideration of several practical factors and, promising outlook. Although studies report high stability for AgNPs under environmental conditions^67,68^ with some even degrading in less than a month,^67^ these characteristics must be weighed against potential ecological risks. For instance, toxic byproducts could form under certain conditions and affect nontarget organisms.^69^

These potential risks, however, are balanced by significant economic and environmental advantages. The proposed green synthesis method, using natural reducing agents, is significantly less expensive than approaches that rely on hazardous and costly chemical compounds.^68,70^ Furthermore, inorganic nanoparticles generally have a lower economic cost than their organic counterparts.^71,72^ A techno-economic analysis will also be necessary to determine the cost-feasibility and scalability of this green approach. Recent studies show different efforts to achieve large-scale manufacturing.^73,74^ The ability to functionalize these nanoparticles with molecules like hesperidin and hesperetin, which can be valorized from industrial citrus waste,^40^ further positions them as a potentially cost-effective and sustainable alternative to current pesticides. Finally, for practical implementation, these nanoparticles should be considered as a component within a broader Integrated Pest Management (IPM) program.^71,75^ Their use could be rotated with reduced-application copper sprays or other biocontrol agents to manage resistance and minimize the overall environmental chemical load, rather than as a standalone replacement.

## Conclusions

Silver nanoparticles functionalized with hesperetin and hesperidin demonstrated potent antibacterial activity against *X. axonopodis* pv. *citri*, with inhibitory concentrations lower than the conventional antibiotic streptomycin. An integrative analysis revealed that the two nanoparticles operate through distinct mechanisms. The AgNP@HSD treatment induced a broad, global stress response in the bacteria, characterized by significant increases in various amino acids and extensive morphological damage to the cell membrane. However, key energy molecules like NAD­(P)H and ATP remained stable, suggesting the bacteria mounted a widespread defensive reaction involving metabolic shifts and membrane remodeling.

In contrast, AgNP@HST appeared to inflict a more targeted form of oxidative damage, evidenced by a significant depletion of both NAD­(P)H and ATP, which point to a collapse of the cell’s energy and redox systems. This more direct and potent mechanism is likely linked to the higher intrinsic bioactivity of hesperetin, which is the aglycone (nonsugar) form of hesperidin. While these findings offer valuable insight into how nanoparticle functionalization can modulate antibacterial mechanisms, further investigation is necessary to fully harness the therapeutic potential and confirm the safety of these promising agents.

## References

[1] Ali S., Hameed A., Muhae-Ud-Din G., Ikhlaq M., Ashfaq M., Atiq M., Ali F., Zia Z. U., Naqvi S. A. H., Wang Y. (2023). Citrus Canker: A Persistent Threat to the Worldwide
Citrus Industry—An Analysis. Agronomy.

[2] Shahbaz E., Ali M., Shafiq M., Atiq M., Hussain M., Balal R. M., Sarkhosh A., Alferez F., Sadiq S., Shahid M. A. (2023). Citrus
Canker Pathogen, Its Mechanism of Infection, Eradication, and Impacts. Plants.

[3] Naqvi S. A. H., Wang J., Malik M. T., Umar U.-U.-D., Ateeq-Ur-Rehman, Hasnain A., Sohail M. A., Shakeel M. T., Nauman M., Hafeez-ur-Rehman, Hassan M. Z., Fatima M., Datta R. (2022). Citrus Canker—Distribution, Taxonomy, Epidemiology, Disease
Cycle, Pathogen Biology, Detection, and Management: A Critical Review
and Future Research Agenda. Agronomy.

[4] Mirzaei-Najafgholi H., Aeini M., Tarighi S., Golmohammadi M. (2021). Comparing
bacterial properties in relation to the virulence factors of Xanthomonas
citri subsp. citri strains and evaluating resistance of subtribe *Citrinae* cultivars to the most virulent strain. J. Plant Pathol..

[5] Dey R., Raghuwanshi R. (2024). An insight
into pathogenicity and virulence gene content
of Xanthomonas spp. and its biocontrol strategies. Heliyon.

[6] Behlau F., Belasque J., Leite R. P., Filho A. B., Gottwald T. R., Graham J. H., Scandelai L. H. M., Primiano I. V., Bassanezi R. B., Ayres A. J. (2021). Relative Contribution
of Windbreak, Copper Sprays, and Leafminer Control for Citrus Canker
Management and Prevention of Crop Loss in Sweet Orange Trees. Plant Dis..

[7] de
Faria I. V. P., da Cunha E. F. F., Freitas M. P. (2025). Exploring the antibacterial
potential of 1,3,4-oxadiazoles against *Xanthomonas axonopodis* pv. *citri* in citrus species through molecular modeling. J. Plant Pathol..

[8] Zhu M., Li Y., Long X., Wang C., Ouyang G., Wang Z. (2022). Antibacterial
Activity of Allicin-Inspired Disulfide Derivatives against *Xanthomonas axonopodis* pv. citri. Int. J. Mol. Sci..

[9] Atiq M., Mazhar H. M. R., Rajput N. A., Ahmad U., Hameed A., Lodhi A. M., Usman M., Nawaz A., Ammar M., Khalid M. (2022). Green Synthesis of Silver and Copper Nanoparticles
from Leaves of Eucalyptus globulus and Assessment of its Antibacterial
Potential Towards *Xanthomonas citri* pv. *citri* Causing Citrus Canker. Appl. Ecol. Environ.
Res..

[10] Silva I. C., Regasini L. O., Petrônio M. S., Silva D. H., Bolzani V. S., Belasque J., Sacramento L. V., Ferreira H. (2013). Antibacterial activity
of alkyl gallates against *Xanthomonas citri* subsp. *citri*. J. Bacteriol..

[11] Jiang S., Tang X., Chen M., He J., Su S., Liu L., He M., Xue W. (2020). Design, synthesis
and antibacterial
activities against *Xanthomonas oryzae* pv. *oryzae*, *Xanthomonas axonopodis* pv. *citri* and *Ralstonia*
*solanacearum* of novel myricetin derivatives containing sulfonamide moiety. Pest Manage. Sci..

[12] Jumpathong J., Suphrom N., Dell B., Khamsuk K., Boonsrangsom T., Poonpaiboonpipat T. (2020). Screening
of Antibacterial Activity of *Goniothalamus
calvicarpa* Extracts against *Xanthomonas axonopodis* pv. *citri*
*in vitro*. Chiang Mai Univ. J. Nat. Sci..

[13] Rodrigues A. S., Batista J. G. S., Rodrigues M. Á.
V., Thipe V. C., Minarini L. A. R., Lopes P. S., Lugão A. B. (2024). Advances
in silver nanoparticles: a comprehensive review on their potential
as antimicrobial agents and their mechanisms of action elucidated
by proteomics. Front. Microbiol..

[14] Basavegowda N., Baek K.-H. (2021). Multimetallic nanoparticles as Alternative
Antimicrobial
Agents: Challenges and Perspectives. Molecules.

[15] Luo L., Huang W., Zhang J., Yu Y., Sun T. (2024). Metal-Based
Nanoparticles as Antimicrobial Agents: A Review. ACS Appl. Nano Mater..

[16] Ribeiro A. I., Dias A. M., Zille A. (2022). Synergistic
Effects Between Metal
Nanoparticles and Commercial Antimicrobial Agents: A Review. ACS Appl. Nano Mater..

[17] Mercan D.-A., Niculescu A.-G., Grumezescu A. M. (2022). Nanoparticles for antimicrobial agents
delivery—an up-to-date review. Int. J.
Mol. Sci..

[18] Goda E. S., Elella M. H. A., Sohail M., Singu B. S., Pandit B., Shafey A. M. E., Aboraia A. M., Gamal H., Hong S. E., Yoon K. R. (2021). N-methylene phosphonic acid chitosan/graphene sheets
decorated with silver nanoparticles as green antimicrobial agents. Int. J. Biol. Macromol..

[19] Alotaibi A. M., Alsaleh N. B., Aljasham A. T., Tawfik E. A., Almutairi M. M., Assiri M. A., Alkholief M., Almutairi M. M. (2022). Silver
Nanoparticle-Based Combinations with antimicrobial agents against
antimicrobial-resistant clinical isolates. Antibiotics.

[20] Dube E., Okuthe G. E. (2025). Silver Nanoparticle-Based
Antimicrobial coatings: Sustainable
strategies for microbial contamination control. Microbiol. Res..

[21] Santos K. S., Barbosa A. M., da Costa L. P., Pinheiro M. S., Oliveira M. B. P. P., Padilha F. F. (2016). Silver nanocomposite biosynthesis:
Antibacterial activity
against multidrug-resistant strains of *Pseudomonas aeruginosa* and *Acinetobacter baumannii*. Molecules.

[22] Mohanta Y. K., Nayak D., Mishra A. K., Chakrabartty I., Ray M. K., Mohanta T. K., Tayung K., Rajaganesh R., Vasanthakumaran M., Muthupandian S., Murugan K., Sharma G., Dahms H.-U., Hwang J.-S. (2022). Green synthesis of endolichenic fungi
functionalized silver nanoparticles: The role in antimicrobial, anti-cancer,
and mosquitocidal activities. Int. J. Mol. Sci..

[23] Choi S.-S., Lee S.-H., Lee K.-A. (2022). A Comparative
Study of Hesperetin,
Hesperidin and Hesperidin Glucoside: Antioxidant, Anti-Inflammatory,
and Antibacterial Activities *in vitro*. Antioxidants.

[24] Li Y. M., Li X. M., Li G. M., Du W. C., Zhang J., Li W. X., Xu J., Hu M., Zhu Z. (2008). *In
vivo* pharmacokinetics of hesperidin are affected by treatment
with glucosidase-like BglA protein isolated from yeasts. J. Agric. Food Chem..

[25] Zare M., Sarkati M. N., Tashakkorian H., Partovi R., Rahaiee S., Rezaei P., Razavi S. A. (2021). Dextran–Hesperetin Conjugate
as a Novel Biocompatible Medicine for Antimicrobial and Anticancer
Applications. J. Polym. Environ..

[26] Zuo J., Liao L., Gao Y., Chen J., Teng J., Zhang W., Wang Y., Sun Y., Liu X. (2025). Antibacterial
effects of cinnamaldehyde and hesperitin on resistant *Glaesserella
parasuis* by suppressing QseBC two-component system. BMC Vet. Res..

[27] Akbar N., Kawish M., Khan N. A., Shah M. R., Alharbi A. M., Alfahemi H., Siddiqui R. (2022). Hesperidin-, Curcumin-,
and Amphotericin
B- Based Nano-Formulations as Potential Antibacterials. Antibiotics.

[28] Pyrzynska K. (2022). Hesperidin:
A Review on Extraction Methods, Stability and Biological Activities. Nutrients.

[29] Attia G. H., Marrez D. A., Mohammed M. A., Albarqi H. A., Ibrahim A. M., Raey M. A. E. (2021). Synergistic Effect
of Mandarin Peels and Hesperidin
with Sodium Nitrite against Some Food Pathogen Microbes. Molecules.

[30] Lee H.-J., Lee S.-H., Hong S.-K., Gil B.-I., Lee K.-A. (2024). *In vitro* Biological Activities of
Hesperidin-Related Compounds
with Different Solubility. Antioxidants.

[31] Di
Minno A., Gelzo M., Caterino M., Costanzo M., Ruoppolo M., Castaldo G. (2022). Challenges in Metabolomics-Based
Tests, Biomarkers Revealed by Metabolomic Analysis, and the Promise
of the Application of Metabolomics in Precision Medicine. Int. J. Mol. Sci..

[32] Alarcon-Barrera J. C., Kostidis S., Ondo-Mendez A., Giera M. (2022). Recent advances in
metabolomics analysis for early drug development. Drug Discovery Today.

[33] Zhou B., Xiao J. F., Tuli L., Ressom H. W. (2012). LC-MS-based metabolomics. Mol.
BioSyst..

[34] Kacerova T., Pires E., Walsby-Tickle J., Probert F., McCullagh J. S. O. (2025). Integrating
NMR and multi-LC-MS-based untargeted metabolomics for comprehensive
analysis of blood serum samples. Anal. Chim.
Acta.

[35] Chen C., Lee D., Yu J., Lin Y., Lin T. (2023). Recent advances in
LC-MS-based metabolomics for clinical biomarker discovery. Mass Spectrom. Rev..

[36] Wishart D.
S., Cheng L. L., Copié V., Edison A. S., Eghbalnia H. R., Hoch J. C., Gouveia G. J., Pathmasiri W., Powers R., Schock T. B., Summer L. W., Uchimiya M. (2022). NMR and Metabolomics—A
Roadmap for the Future. Metabolites.

[37] Moco S. (2022). Studying Metabolism
by NMR-Based Metabolomics. Front. Mol. Biosci..

[38] Pang Z., Lu Y., Zhou G., Hui F., Xu L., Viau C., Spigelman A. F., MacDonald P. E., Wishart D. S., Li S., Xia J. (2024). MetaboAnalyst
6.0: towards a unified platform for metabolomics data
processing, analysis and interpretation. Nucleic
Acids Res..

[39] Omidfar F., Gheybi F., Davoodi J., Amirinejad M., Badiee A. (2023). Nanophytosomes of hesperidin and of hesperetin: Preparation,
characterization, and *in vivo* evaluation. Biotechnol. Appl. Biochem..

[40] de
Castro S. C., Stanisic D., Tasic L. (2024). Sequential extraction
of hesperidin, pectin, lignin, and cellulose from orange peels: towards
valorization of agro-waste. Biofuels, Bioprod.
Biorefin..

[41] Binkowska I. (2020). Hesperidin:
synthesis and characterization of bioflavonoid complex. SN Appl. Sci..

[42] Alipour M., Sharifi S., Samiei M., Shahi S., Aghazadeh M., Dizaj S. M. (2023). Synthesis, characterization, and
evaluation of hesperetin
nanocrystals for regenerative dentistry. Sci.
Rep..

[43] Fathi M., Varshosaz J. (2013). Novel hesperetin
loaded nanocarriers for food fortification:
Production and characterization. J. Funct. Foods.

[44] Menichetti A., Mavridi-Printezi A., Mordini D., Montalti M. (2023). Effect of Size, Shape
and Surface Functionalization on the Antibacterial Activity of Silver
Nanoparticles. J. Funct. Biomater..

[45] Ahmad F., Salem-Bekhit M. M., Khan F., Alshehri S., Khan A., Ghoneim M. M., Wu H.-F., Taha E. I., Elbagory I. (2022). Unique Properties
of Surface-Functionalized Nanoparticles for Bio-Application: Functionalization
Mechanisms and Importance in Application. Nanomaterials.

[46] Luceri A., Francese R., Lembo D., Ferraris M., Balagna C. (2023). Silver Nanoparticles:
Review of Antiviral Properties, Mechanism of Action and Applications. Microorganisms.

[47] Restrepo C. V., Villa C. C. (2021). Synthesis of silver
nanoparticles, influence of capping
agents, and dependence on size and shape: A review. Environ. Nanotechnol., Monit. Manage..

[48] Aiad I., Marzouk M. I., Shaker S. A., Ebrahim N. E., Abd-Elaal A. A., Tawfik S. M. (2017). Antipyrine cationic
surfactants capping silver nanoparticles
as potent antimicrobial agents against pathogenic bacteria and fungi. J. Mol. Liq..

[49] Flieger J., Franus W., Panek R., Szymańska-Chargot M., Flieger W., Flieger M., Kołodziej P. (2021). Green Synthesis
of Silver Nanoparticles Using Natural Extracts with Proven Antioxidant
Activity. Molecules.

[50] Parvekar P., Palaskar J., Metgud S., Maria R., Dutta S. (2020). The minimum
inhibitory concentration (MIC) and minimum bactericidal concentration
(MBC) of silver nanoparticles against *Staphylococcus aureus*. Biomater. Invest. Dent..

[51] Krishna R., Arumugam V., Vasaviah S. K. (2015). The MIC
and MBC of Silver Nanoparticles
against *Enterococcus faecalis* - A Facultative Anaerobe. J. Nanomed. Nanotechnol..

[52] Hamouda R. A., Yousuf W. E., Abdeen E. E., Mohamed A. (2019). Biological
and Chemical
Synthesis of Silver Nanoparticles: Characterization, MIC and Antibacterial
Activity against Pathogenic Bacteria. J. Chem.
Pharm. Res..

[53] Almukainzi M., El-Masry T. A., El Zahaby E. I., El-Nagar M. M. F. (2024). Chitosan/Hesperidin
Nanoparticles for Sufficient, Compatible, Antioxidant, and Antitumor
Drug Delivery Systems. Pharmaceuticals.

[54] Zhao Z.-y., Li P.-J., Xie R.-S., Cao X.-Y., Su D.-L., Shan Y. (2022). Biosynthesis of silver
nanoparticle composites based on hesperidin
and pectin and their synergistic antibacterial mechanism. Int. J. Biol. Macromol..

[55] Aragaw W. W., Negatu D. A., Bungard C. J., Dartois V. A., Marrouni A. E., Nickbarg E. B., Olsen D. B., Warrass R., Dick T. (2024). Pharmacological
validation of dihydrofolate reductase as a drug target in Mycobacterium
abscessus. Antimicrob. Agents Chemother..

[56] Vassiliades S. V., Borges L. G., Giarolla J., Parise-Filho R. (2023). Folate Pathway
Inhibitors, An Underestimated and Underexplored Molecular Target for
New Anti-tuberculosis Agents. Mini-Rev. Med.
Chem..

[57] Chain C., Sheehan J. P., Xu X., Ghaffari S., Godbole A., Kim H., Freundlich J. S., Rabinowitz J. D., Gitai Z. (2024). A folate inhibitor exploits metabolic
differences in Pseudomonas
aeruginosa for narrow-spectrum targeting. Nat.
Microbiol..

[58] Zhang R., Piao M. J., Kim K. C., Kim A. D., Choi J. Y., Choi J., Hyun J. W. (2012). Endoplasmic reticulum
stress signaling
is involved in silver nanoparticles-induced apoptosis. Int. J. Biochem. Cell Biol..

[59] Durán N., Durán M., de Jesus M. B., Seabra A. B., Fávaro W. J., Nakazato G. (2016). Silver nanoparticles: A new view on mechanistic aspects
on antimicrobial activity. Nanomedicine.

[60] McQuillan J. S., Groenaga Infante H., Stokes E., Shaw A. M. (2012). Silver nanoparticle
enhanced silver ion stress response in *Escherichia
coli* K12. Nanotoxicol.: Nanotechnol.,
Biol. Med..

[61] Flores-López L. Z., Espinoza-Gómez H., Somanathan R. (2019). Silver nanoparticles:
Electron transfer, reactive oxygen species, oxidative stress, beneficial
and toxicological effects. J. Appl. Toxicol..

[62] Quinteros M. A., Aristizábal V. C., Dalmasso P. R., Paraje M. G., Páez P. L. (2016). Oxidative
stress generation of silver nanoparticles
in three bacterial genera and its relationship with the antimicrobial
activity. Toxicol. In Vitro.

[63] Campo-Beleño C., Villamizar-Gallardo R. A., López-Jácome L. E., González E. E., Muñoz-Carranza S., Franco B., Morales-Espinosa R., Coria-Jimenez R., Franco-Cendejas R., Hernández-Durán M., Lara-Martínez R., Jiménez-García L. F., Fernández-Presas A. M., García-Contreras R. (2022). Biologically
synthesized silver nanoparticles
as potent antibacterial effective against multidrug-resistant *Pseudomonas aeruginosa*. Lett. Appl.
Microbiol..

[64] M S., N R. P., Rajendrasozhan S. (2023). Bacterial redox response factors
in the management of environmental oxidative stress. World J. Microbiol. Biotechnol..

[65] Krapp A. R., Humbert M. V., Carrillo N. (2011). The soxRS
response of *Escherichia coli* can be
induced in the absence of
oxidative stress and oxygen by modulation of NADPH content. Microbiology.

[66] Nikel P. I., Fuhrer T., Chavarría M., Sánchez-Pascuala A., Sauer U., de Lorenzo V. (2021). Reconfiguration of metabolic fluxes
in *Pseudomonas putida* as a response to sub-lethal
oxidative stress. ISME J..

[67] Levard C., Hotze E. M., Lowry G. V., Brown G. E. (2012). Environmental Transformations of
Silver Nanoparticles: Impact on
Stability and Toxicity. Environ. Sci. Technol..

[68] Osman A. I., Zhang Y., Farghali M., Rashwan A. K., Eltaweil A. S., El-Monaem E. M. A., Mohamed I. M. A., Badr M. M., Ihara I., Rooney D. W., Yap P.-S. (2024). Synthesis of green nanoparticles
for energy, biomedical. Environ. Chem. Lett..

[69] Kim H.-A., Choi Y. J., Kim K.-W., Lee B.-T., Ranvill J. F. (2012). Nanoparticles
in the environment: stability and toxicity. Rev. Environ. Health.

[70] Omran, B. A. Biosynthesized Nanomaterials via Processing of Different Plant Parts (Phytonanotechnology) and Biovalorization of Agro-Industrial Wastes to Nano-Sized Valuable Products. In Nanotechnology in the Life Sciences; Springer, 2020; pp 145–184.

[71] Athanassiou C. G., Kavallieratos N. G., Benelli G., Losic D., Rani P. U., Desneux N. (2018). Nanoparticles for pest control: Current status and
future perspectives. J. Pest Sci..

[72] Rehmanullah, N. ; Muhammad, Z. ; Inayat, N. ; Majeed, A. Application of Nanoparticles in Agriculture as Fertilizers and Pesticides: Challenges and Opportunities. In New Frontiers in Stress Management for Durable Agriculture; Springer, 2020; pp 281–293.

[73] Fernandes, C. ; Jathar, M. ; Sawant, B. K. S. ; Warde, T. Scale-up of nanoparticle manufacturing process. In AAPS Introductions in the Pharmaceutical Sciences; Springer, 2023; Vol. 13, pp 173–203.

[74] Piccinino D., Capecchi E., Delfino I., Crucianelli M., Conte N., Avitabile D., Saladino R. (2021). Green and Scalable
Preparation of Colloidal Suspension of Lignin Nanoparticles and Its
Application in Eco-friendly Sunscreen Formulations. ACS Omega.

[75] Subramaniam J., Murugan K., Panneerselvam C., Kovendan K., Madhiyazhagan P., Kumar P. M., Dinesh D., Chandramohan B., Suresh U., Nicoletti M., Higuchi A., Hwang J. S., Kumar S., Alarfaj A. A., Munusamy M. A., Messing R. H., Benelli G. (2015). Eco-friendly control
of malaria and arbovirus vectors
using the mosquitofish *Gambusia affinis* and ultra-low
dosages of *Mimusops elengi*-synthesized silver nanoparticles:
towards an integrative approach?. Environ. Sci.
Pollut Res. Int..

